# Deciphering Depression: Epigenetic Mechanisms and Treatment Strategies

**DOI:** 10.3390/biology13080638

**Published:** 2024-08-20

**Authors:** Alaa A. A. Aljabali, Almuthanna K. Alkaraki, Omar Gammoh, Murtaza M. Tambuwala, Vijay Mishra, Yachana Mishra, Sk. Sarif Hassan, Mohamed El-Tanani

**Affiliations:** 1 Faculty of Pharmacy, Department of Pharmaceutics & Pharmaceutical Technology, Yarmouk University, Irbid 21163, Jordan; 2 Department of Biological Sciences, Faculty of Science, Yarmouk University, Irbid 21163, Jordan; alkaraki@yu.edu.jo; 3 Department of Clinical Pharmacy and Pharmacy Practice, Faculty of Pharmacy, Yarmouk University, P.O. Box 566, Irbid 21163, Jordan; omar.gammoh@yu.edu.jo; 4 College of Pharmacy, Ras Al Khaimah Medical and Health Sciences University, Ras Al Khaimah P.O. Box 11172, United Arab Emirates; mtambuwala@gmail.com (M.M.T.); eltanani@rakmhsu.ac.ae (M.E.-T.); 5 School of Pharmaceutical Sciences, Lovely Professional University, Phagwara 144411, Punjab, India; vijaymishra2@gmail.com; 6 School of Bioengineering and Biosciences, Lovely Professional University, Phagwara 144411, Punjab, India; yachanamishra@gmail.com; 7 Department of Mathematics, Pingla Thana Mahavidyalaya, Maligram, Paschim Medinipur 721140, West Bengal, India; sksarifhassan@pinglacollege.ac.in

**Keywords:** epigenetics, antidepressant drugs, DNA methylation, non-coding RNAs, personalized medicine, biomarkers, histone modifications

## Abstract

**Simple Summary:**

Epigenetics plays a significant role in understanding depression, which is considered one of the most complex mental health disorders. Epigenetics includes all changes in gene expression that do not involve any modifications to DNA sequences. In depression, epigenetic changes may affect the activation or suppression of specific genes involved in mood regulation. Scientists have attempted to determine how specific environmental factors, such as stress or trauma, can trigger epigenetic changes that are closely linked to gene function. By studying these mechanisms, researchers have developed effective treatments and interventions for depression. This discovery not only provides a better understanding of the biological basis of depression, but also suggests new approaches for highly personalized treatments that may be available to individuals suffering from this widespread mental health disorder in the near future.

**Abstract:**

Depression, a significant mental health disorder, is under intense research scrutiny to uncover its molecular foundations. Epigenetics, which focuses on controlling gene expression without altering DNA sequences, offers promising avenues for innovative treatment. This review explores the pivotal role of epigenetics in depression, emphasizing two key aspects: (I) identifying epigenetic targets for new antidepressants and (II) using personalized medicine based on distinct epigenetic profiles, highlighting potential epigenetic focal points such as DNA methylation, histone structure alterations, and non-coding RNA molecules such as miRNAs. Variations in DNA methylation in individuals with depression provide opportunities to target genes that are associated with neuroplasticity and synaptic activity. Aberrant histone acetylation may indicate that antidepressant strategies involve enzyme modifications. Modulating miRNA levels can reshape depression-linked gene expression. The second section discusses personalized medicine based on epigenetic profiles. Analyzing these patterns could identify biomarkers associated with treatment response and susceptibility to depression, facilitating tailored treatments and proactive mental health care. Addressing ethical concerns regarding epigenetic information, such as privacy and stigmatization, is crucial in understanding the biological basis of depression. Therefore, researchers must consider these issues when examining the role of epigenetics in mental health disorders. The importance of epigenetics in depression is a critical aspect of modern medical research. These findings hold great potential for novel antidepressant medications and personalized treatments, which would significantly improve patient outcomes, and transform psychiatry. As research progresses, it is expected to uncover more complex aspects of epigenetic processes associated with depression, enhance our comprehension, and increase the effectiveness of therapies.

## 1. Introduction

Depression is a common and complex mental disorder that significantly affects individuals worldwide [1]. There are several types of depression, and among them, major depressive disorder (MDD) is one of the most common mental health disorders. This causes persistent sadness and a lack of interest, affecting people in various ways, both emotionally and physically, in their daily lives. Other types of depression, besides MDD, include dysthymic and bipolar disorders [2,3]. Classical genetic studies have traditionally focused on understanding the genetic basis of this condition. Emerging epigenetic studies have provided new perspectives beyond classical genetics. These studies shed light on the intricate mechanisms of gene regulation that contribute to the development of depression [4]. Epigenetics refers to the study of heritable changes in gene expression that do not involve alterations in DNA sequences. This field encompasses various mechanisms, including DNA methylation, histone modification, and non-coding RNA expression, all of which play roles in gene regulation. Therefore, epigenetic processes involve dynamic interactions between genes and the environment at a cellular, individual, and population level [5,6,7]. Research on depression has identified abnormal DNA methylation patterns in key genes related to neurodevelopment and neurotransmission, particularly those involved in serotonin and dopamine pathways. For example, epigenetic changes in the serotonin transport gene (SLC6A4) are associated with vulnerability to depression and treatment response [8]. Furthermore, alterations in DNA methylation of the brain-derived neurotrophic factor (BDNF) gene, a crucial gene involved in neuroplasticity, have been observed in the pathophysiology of depression, suggesting a potential mechanism for stress-induced mood changes [9]. Recent studies have also highlighted the role of post-transcriptional regulators, such as microRNAs (miRNAs) in depression [10,11]. Aberrant miRNA expression has been implicated in neuroinflammation, synaptic plasticity, and stress response pathways, potentially serving as a biomarker for diagnostic and therapeutic purposes.

Acetylation and methylation of histones regulate the expression of genes involved in synaptic adaptability and stress response. These changes in chromatin conformation and transcriptional activity are influenced by environmental factors [12]. Targeting enzymes involved in histone modification pathways may offer new therapeutic avenues for restoring the gene expression balance in depression [13]. Despite the progress made in integrating epigenetics into depression research, several challenges remain. Ethical considerations arise regarding the use of human brain tissue and the need for longitudinal sampling [14,15]. Nevertheless, advancements in technology have enabled the profiling of complex epigenetic modifications in the brain, providing valuable insights. Longitudinal studies are crucial to determine whether these alterations are causative factors or consequences of the disorder.

The ethical implications of epigenetics in depression are complex. Furthermore, this also raises ethical concerns at an individual and societal level [16]. The fact that epigenetic modifications are heritable adds another layer of complexity, whereby there may be long-term health effects in future generations due to current environmental exposure [17]. Additionally, using epigenetic risk-predictive screening for depression presents challenges in obtaining informed consent and effectively communicating complex risk information [16]. Further research is needed to understand how present actions affect the epigenome and well-being of descendants [17]. These are sensitive issues in a very vulnerable population, inundated with special exposure risks and the potential for further marginalization, in the population of refugees and asylum seekers [18]. In the context of assisted reproductive technologies, ethical considerations are heightened, given the significant implications for all parties involved [19].

Therefore, the ethical, legal, and social implications of returning the results and incidental findings of epigenetic research should be thoroughly deliberated, which should be the standard within the research community [18]. The ethical landscape of epigenetics in depression is complex, even in child psychiatry, in which epigenetic research presents both potential contributions and challenges. Some researchers have expressed concerns about the non-medical application of epigenetics and have emphasized the need for ethical reflection. Moreover, the social implications of epigenetic research, as portrayed in the media, should be critically evaluated to ensure responsible and informed communication [19]. Ethical concerns in the field of epigenetics and depression are extensive and encompass issues related to intergenerational effects, research involving vulnerable populations, assisted reproduction technologies, return of research results, and societal implications. These ethical considerations have not received sufficient attention, highlighting the importance of conducting research on epigenetic depression with a sense of responsibility toward individuals and communities [19,20].

Epigenetics has emerged as an exciting frontier in the study of gene regulation and its implications for human health and disease. Among the numerous processes that occur in living organisms, mental health has led to the exploration of epigenetic mechanisms (EMs). This critical review highlights the relationship between epigenomics and mental health, emphasizing recent evidence on the epigenetics of psychiatric disorders [21,22].

To avoid overlaying the biomedical view of depression, existential reflection may resemble conditions that simulate depression; however, it may not necessarily be pathological. This understanding is supported by research examining existential aspects of mental health. Indeed, one study suggested that existential conditions may be closely connected to depression because they raise existential concerns that are reflected in the symptoms [23,24]. Critical literature has emerged that focuses on the roles of existential components in caring for individuals with stress-related disorders, demonstrating that these diseases can have long-lasting negative effects on well-being [25].

**Figure 1:**
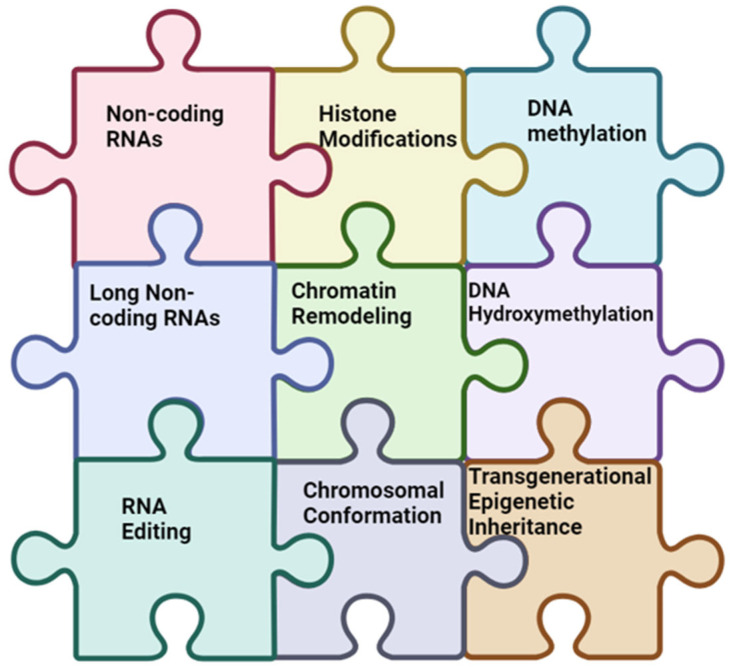
Deciphering the epigenetic puzzle of depression: this intricate puzzle highlights the interconnected pathways linking EMs to depression. Key elements include non-coding RNAs, histone modifications, DNA methylation, long non-coding RNAs orchestrating chromatin remodeling, DNA hydroxy methylation, RNA editing, chromosomal conformational changes, and the transgenerational inheritance of epigenetic modifications. Understanding the dynamic interplay between these factors provides insight into the epigenetic landscape associated with depression and its potential transgenerational impact on mental health.

For instance, Restifo et al. suggested that existential depression is a distinct diagnosis, but does not fit the typical view of depression as a pathological condition [26]. This perspective allows for considering depression not only as a disorder but also as an opportunity to develop a new, realistic perception of life. After all, something cannot cause existential threats or trigger negative emotions if a defense mechanism is not required. This possibility highlights the psychological nature of existential threats and negative emotions rather than their physiological basis [27]. Regarding Parkinson’s disease (PD), an established association highlights the importance of monitoring changes in neurochemicals and identifying clinical signs, such as the asthenic “mask” in a population [27,28]. Additionally, the fact that depressive syndrome in PD is partially influenced by serotoninergic pathology blurs the line between neurological and mood disorders [29]. The states of existential and reflective depression do not fit the typical pathological paradigms of depression. An analysis of existential-oriented features and an existential–philosophical approach to understanding mental health revealed that existential reflections can disrupt emotional well-being in ways that go beyond the usual diagnostic criteria for depression. This review explores the complex relationship between epigenetics and mental health by examining recent discoveries that have revealed epigenetic aspects of psychiatric disorders as reflected in Figure 1. As scientific knowledge progresses, the potential for more effective and personalized approaches to manage depression has become increasingly promising.

### Background and Significance

Mental health disorders, such as anxiety, depression, schizophrenia, and bipolar disorder, significantly impact millions globally [30]. Traditionally, research has focused on unraveling the genetic underpinnings of these complex conditions. However, a growing body of evidence indicates that EMs also play a pivotal role in modulating gene activation associated with brain maturation, neural plasticity, and emotion regulation [31,32]. A comprehensive understanding of epigenetics in mental health could lead to groundbreaking therapeutic interventions and personalized treatment.

Epigenetics encompasses heritable modifications in gene expression without alterations in DNA sequences. The key mechanisms include DNA methylation, histone modification, and non-coding RNAs. DNA methylation involves the addition of methyl groups to cytosine residues, which influences gene expression by impeding transcription factors [33,34]. Histone modifications, including acetylation, methylation, and phosphorylation, alter chromatin structure and accessibility, thereby shaping gene functions. Non-coding RNAs, particularly miRNAs, play crucial roles in the post-transcriptional control and fine-tuning of gene expression [35]. During early brain development, epigenetic processes guide neural stem cell differentiation, thereby directing the formation of distinct brain regions and neural circuits. Epigenetic modifications are essential for synaptic plasticity, a fundamental process in learning and memory. Dysregulation of these processes is linked to neurodevelopmental disorders such as autism spectrum disorder (ASD) and intellectual disabilities, highlighting the intricate interplay between epigenetics and mental health [36,37]. Acute or chronic stress profoundly affects mental well-being, and can precipitate mood disorders and anxiety-related conditions. Stress-induced epigenetic modifications occur in crucial brain regions involved in stress regulation and emotion processing, such as the hippocampus and amygdala. Understanding how epigenetics mediates the connection between stress and mental health may unveil new therapeutic avenues targeting the lasting effects of stress on the brain [38].

Recent studies have identified specific epigenetic signatures that are associated with various psychiatric disorders. For instance, aberrant DNA methylation patterns in genes related to synaptic function have been observed in individuals with schizophrenia, suggesting an association between epigenetic changes and the underlying mechanisms of the disorder. Similarly, alterations in histone modifications have been implicated in mood disorders and are promising targets for novel treatments [39]. Advancements in epigenetic research present unprecedented opportunities for understanding and addressing mental health disorders. Integrating epigenomic data with other omics approaches and advanced bioinformatics tools may reveal the previously unknown molecular networks underlying psychiatric conditions. Identifying novel epigenetic biomarkers and understanding their functional relevance could pave the way for personalized treatment strategies based on an individual’s unique epigenetic profile [40]. In conclusion, the rapidly evolving field of epigenetics has a significant potential for elucidating the complex interactions between genetic susceptibility and environmental influences on mental health disorders. Exploring epigenetic intricacies in the brain reveals novel pathways for understanding the origins and mechanisms of, and therapeutic strategies for, psychiatric disorders. As we navigate this uncharted territory and decipher the epigenetic intricacies of the mind, we stand on the cusp of transforming our approach to mental well-being, ultimately enhancing the lives of countless individuals worldwide [32,41].

## 2. Epigenetic Mechanisms (EMs)

### 2.1. DNA Methylation

Through this modification, long-term changes in gene expression patterns can be induced [42]. This addition can result in transcriptional silencing by either occluding access to transcription factors or recruiting methyl-CpG binding proteins, eventually leading to a repressive chromatin state. DNA methylation patterns have been studied in major depression, both with and without the disorder (patients during the remission phase of MDD), to elucidate the involvement of EMs in the underlying processes of depression [43,44,45]. There is now substantial evidence linking changes in DNA methylation to the development of major depression and its associated behavioral symptoms [46,47]. The epigenetic nature of depression, as evidenced by changes in the DNA methylation of specific genes and genomic regions, is strongly associated with cardiometabolic factors [48].

Abnormal methylation of certain alleles within the six CpG sites has been identified as a contributor to atypical BDNF expression and has been implicated in depression, suggesting a potential connection between epigenetic regulation and MDD [49]. These changes can be seen in promoters, regulatory elements such as enhancers, and genes involved in important neurobiological processes [50]. Alterations in normal patterns of gene expression caused by changes in DNA methylation can contribute to the development and progression of depressive symptoms. EMs, including histone modifications, non-coding RNA, and DNA methylation, play a role in regulating gene expression [51,52].

A growing body of research has indicated a correlation between DNA methylation and depression. Over-methylation of certain genes, such as *BDNF*, *NR3C1*, and *SLC6A4*, has been identified as a marker of increased susceptibility to depression [53]. Additionally, changes in methylation at specific sites have been associated with the involvement of genes, such as *YOD1*, *UGT8*, *FNDC3B*, and *SLIT2*, in late life depression. However, it is important to recognize that inconsistencies are frequently encountered in this field because of the significant heterogeneity in study methodologies [52]. Increased gene transcription was observed in a larger sample of monozygotic twin pairs, with varying methylation levels in the promoter region of the serotonin transporter gene. Furthermore, these twins showed variations in depressive symptoms, indicating a potential connection between methylation status and depressive outcomes [54].

DNA methyltransferases (DNMTs) play a key role in DNA methylation. DNA methylation is a common form of epigenetic change. Studies have shown that some DNMTs are overexpressed in patients with major depression. Studies have suggested that high levels of DNMTs are linked to stress-mediated epigenetic changes that affect depression [55]. These findings highlight the potential of DNMTs as a therapeutic target in epigenetic depression [56].

### 2.2. Histone Modifications

Evidence shows histone modifications, along with DNA methylation and microRNAs, are intrinsic EMs involved in psychotic diseases including depression [57]. In animal models of chronic stress-induced depression, histone changes have been associated with alterations in the gene expression responsible for neurotransmitter synthesis [58]. Furthermore, studying core histone modifications has gained interest in understanding the mechanisms involved in MDD and treatment interventions. Changes in histone acetylation and methylation in MDD have been identified as important factors that determine disease severity and treatment response [13]. Although animal models have demonstrated the role of histone alterations in depression, further whole-genome profiling of these alterations in various brain regions is required to understand the exact genes and pathways involved in depression and antidepressant treatment responses [1]. Understanding the role of histone modifications in depression could provide valuable insights into its pathophysiology and potential targets for therapeutic interventions [57].

The exploration of histone modifications and their roles in gene regulation has revealed complex epigenetic communication that orchestrates cellular processes. This section presents a comprehensive overview of the findings, analysis, and critical evaluation of histone modifications and their impact on gene expression [59,60]. Histone modifications, including methylation, acetylation, phosphorylation, and ubiquitination, occur at specific amino acid sites within histone tails. These modifications have the potential to affect the accessibility of the underlying DNA to the transcriptional machinery [61,62]. Research has demonstrated the importance of histone modification in gene regulation. For example, histone acetylation, facilitated by histone acetyltransferases (HATs), promotes transcriptional activation by neutralizing the positive charge of histones and loosening chromatin structure. Conversely, histone deacetylases (HDACs) remove acetyl groups, leading to transcriptional suppression. The methylation of histone residues, such as lysine and arginine, can have varying effects on gene expression, depending on the location and extent of methylation [63,64].

The mechanisms governing their functions and interactions are currently under investigation. Inconsistencies arise regarding the specific histone markers and their influence on gene expression. For instance, although trimethylation of lysine 4 on histone H3 (H3K4me3) is commonly associated with transcriptional activation, recent studies have identified cases in which H3K4me3 is found in regions with suppressed transcription, indicating the necessity for a more nuanced interpretation of its role [59,65,66]. The interplay between histone modifications adds complexity to the regulatory landscape. A combination of multiple histone modifications can create distinct binding patterns for effector proteins and establish specific chromatin states. However, our understanding of the functional implications and the hierarchical nature of these combinations remains limited [67]. It is important to acknowledge the limitations of the existing scholarly literature, as many studies concentrate on individual histone modifications and rely on correlation rather than causality. This makes it challenging to establish a relationship between histone modification and gene expression [68,69]. In conclusion, the reported findings, analysis, and critical evaluation of histone modifications in gene regulation demonstrate their indispensable roles in shaping chromatin structure and orchestrating gene expression programs [70,71]. The field continues to evolve rapidly, with ongoing investigations aimed at deciphering the intricacies of histone modifications, their interplay, and their functional consequences. By embracing opposing viewpoints and acknowledging the limitations of the existing literature, future research can pave the way for a more comprehensive understanding of the epigenetic language encoded by histone modifications [72,73].

### 2.3. Non-Coding RNAs

Long non-coding RNAs (lncRNAs) act as key regulators of various EMs and influence gene regulation at multiple levels. Some lncRNAs are specifically expressed in certain cell types and developmental stages [74,75]. These lncRNAs have been reported to be involved in diverse cellular functions related to chromatin dynamics and gene silencing. For instance, the dysregulation of the long non-coding RNA HOTAIR has been observed in several cases of depression, highlighting its crucial role in gene regulation [76,77]. Existing research suggests that lncRNAs interact with the epigenetic machinery by aiding the deposition of chromatin-associated proteins and facilitating changes in gene expression. Studies have indicated that lncRNAs play significant roles in cellular processes such as tumor initiation, metastasis, and drug resistance. Additionally, recent studies have highlighted the involvement of lncRNAs in various diseases through mechanisms that regulate DNA methylation and histone modification [78,79,80]. This finding is particularly surprising considering common human pathologies, such as Alzheimer’s disease and multiple myeloma. The interplay between lncRNAs and epigenetic factors appears to underlie their contribution to pathogenicity.

LncRNAs may play the same role in the epigenetic regulation of depression. Some lncRNAs that affect gene expression in depression may impact genes associated with depression. For example, HOTAIR can influence genes related to mood regulation and stress responses. HOTAIR binds to the polycomb repressive complex 2, as has been reported in depression research. These genes include BDNF, SLC6A4, HTR1A, FKBP5, NR3C1, COMT, TPH2, and MAOA [81,82,83]. BDNF plays a role in neuroplasticity, and SLC6A4 and HTR1A are critical for serotonin signaling. FKBP5 and NR3C1 are the core elements that control stress response. COMT plays a role in dopamine metabolism, TPH2 in serotonin synthesis, and MAOA in the degradation of monoamine neurotransmitters [78,84,85,86].

## 3. Epigenetic Mechanisms in Depression

### 3.1. DNA Methylation and Depression

DNMT inhibitors are used to treat cancer and depression and also show great promise. They can reactivate silenced tumor suppressor genes by blocking DNMTs, creating opportunities for the treatment of depression. Additionally, researchers have studied DNMT inhibitors in combination therapies to enhance other treatments, such as PARP inhibitors, to improve depression treatment [87].

**Figure 2:**
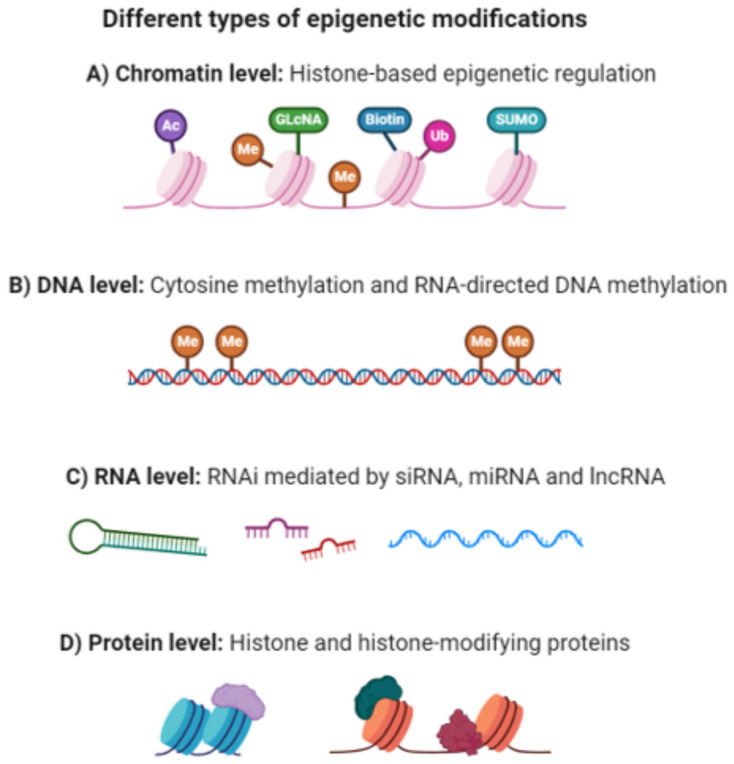
Comprehensive overview of epigenetic modifications: The schematic illustrates the diverse types of epigenetic modifications that occur at various levels within a cellular context. The depicted layers include chromatin-level modifications, such as histone acetylation and methylation, DNA-level modifications, DNA methylation, and RNA-level modifications, highlighting processes such as RNA methylation. Understanding these intricate EM is essential to unraveling the complex regulation of gene expression and cellular functions. Abbreviations used in the figure: Me (methylation), Ac (acetylation), CoA (coenzyme A), SAM (S-adenosylmethionine), GLcNAc (N-acetylglucosamine), Ub (ubiquitination), biotin (a coenzyme for carboxylase enzymes), SUMO (Small Ubiquitin-like Modifier), RNAi (RNA interference), siRNA (small interfering RNA), miRNA (microRNA), and lncRNA (long non-coding RNA). Images were generated using Biorender.com.

DNMT inhibitors are promising therapeutic agents that may alter tumor responses to chemotherapy and radiation therapy [88]. By altering the activity of these enzymes, harmful methylation patterns can be reversed, reducing the symptoms of depression [88,89,90,91]. Understanding DNMTs will aid in research on treatment effectiveness as shown in Figure 2.

### 3.2. Histone Modifications and Depression

Histone modifications, particularly H3K9 trimethylation, have been linked to the control of Crhr1 in the hypothalamus, potentially influencing depression [92]. Similarly, there have been reports suggesting that the onset of MDD is linked to histone modifications, which have potential implications for therapeutic interventions. Electroconvulsive seizures (ECSs) have shown promise in the treatment of depression, potentially by modulating gene expression via histone modifications [93]. Additionally, the dysregulation of synapsin genes, modulated by histone modifications, has been associated with mood disorders including depression [94]. Collectively, these findings underscore the significance of histone modifications in the pathophysiology and management of depression.

### 3.3. Non-Coding RNAs and Depression

These lncRNAs have been shown to participate in various cellular functions associated with chromatin dynamics and gene silencing. The current literature indicates that the mechanisms of action of lncRNAs are exerted through association with epigenetic machinery, facilitating the deposition of chromatin-associated proteins, mostly for the implementation of changes in gene expression. It has been shown that lncRNAs participate in cellular processes, including tumor initiation, metastasis, and drug resistance. More recently, specific studies have revealed that some lncRNAs are involved in various diseases via mechanisms that control DNA methylation and histone modifications [95,96]. This is quite surprising with respect to common human pathologies such as Alzheimer’s disease and multiple myeloma. Pathogenicity is attributed to the interplay between long non-coding RNAs (lncRNAs) and epigenetic factors.

**Figure 3:**
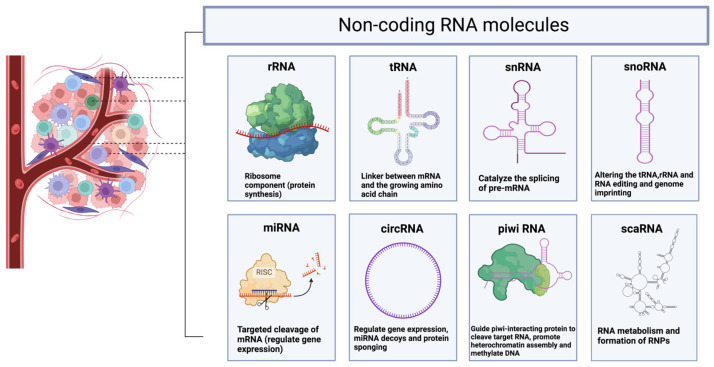
Representation of non-coding RNA molecules, including rRNA (ribosomal RNA), tRNA (transfer RNA), snRNA (small nuclear RNA), snoRNA (small nucleolar RNA), miRNA (microRNA), circRNA (circular RNA), piwiRNA, and scRNA (small cytoplasmic RNA). The images were generated using Biorender.com.

The epigenetic control of lncRNAs may be affected in cases of depression. Certain lncRNAs controlling the genes involved in the development of depression could conceivably influence those related to depression as shown in Figure 3. For instance, HOTAIR is an lncRNA that has been shown to play a role in chromatin remodeling and gene silencing during depression and can influence genes related to mood regulation and stress responses in a very similar vein. There is already evidence from studies of cancer that HOTAIR interacts with the chromatin-modifying complex PRC2 to silence genes encoding tumor suppressors. This could provide a parallel mechanism by which lncRNAs regulate the neurobiological genes involved in depression [78,97,98].

Another lncRNA, NEAT1, controls the genes of signaling pathways via its interaction with regulatory elements located at a distance [80,99]. Such interactions also seem relevant for depression, in which NEAT1 may modulate neuronal gene expression and synaptic function.

LncRNAs have been reported to play key roles in EM and have vital implications in the regulation of gene expression. Their interactions with chromatin modifiers and their involvement in several biological phenomena underlie the complexity of their regulatory functions. It is important to understand these intricate interactions to elucidate the impact of lncRNAs on health and disease [100]. Specific lncRNAs associated with EMs included ANRIL, XIST, HOTAIR, KCNQ1OT1, RMST, CASC8, RP11-65J3.1, SNHG12, CASC11, HOTAIRM1, GAS5, HOTTIP, HAS2AS1, HOXD-AS1, and NEAT1 [82,100,101,102,103]. Therefore, they are among the main epigenetic regulators. This also extends to ANRASSF1, a co-recruited function that expresses and replicates cell proliferation via the polycomb repressive complex 2 at most of the gene promoters. Another example is NEAT1, which regulates genes in signaling pathways by accessing and interacting with dispersed regulatory elements, whose activity, in turn, may not be modulated by the efficiency of these target genes. In summary, taking these examples together, a role for lncRNAs was observed epigenetically by recruiting histone-modifying enzymes to regulate chromatin structure modulation. It is important to understand how lncRNAs participate in various biological processes and diseases [78,97,104]. These studies revealed the role of lncRNAs in depression.

They are involved in several mechanisms that contribute to depression through the regulation of neurotransmitters, neurotrophic factors, and synaptic functions. LncRNAs may also function in cooperation with inflammatory pathways and epigenetic modifications that play a crucial role in the development of depressive disorders [83,105]. Notably, these lncRNAs were associated with altered DNA methylation.

LncRNAs play significant roles in EM and have important implications in gene regulation. Their interactions with chromatin modifiers and involvement in various biological phenomena highlight the complexity of their regulatory functions. Understanding these intricate interactions is crucial for understanding the impact of lncRNAs on health and disease [106].

Some specific lncRNAs associated with EMs include ANRIL, XIST, HOTAIR, KCNQ1OT1, RMST, CASC8, RP11-65J3.1, SNHG12, CASC11, HOTAIRM1, GAS5, HOTTIP, HAS2-AS1, HOXD-AS1, and NEAT1, among others [107]. These lncRNAs not only show associations, but also recruit and interact with epigenetic modifiers at specific loci. They have been shown to interact with chromatin remodeling complexes to influence chromatin conformational patterns, thereby modulating gene expression [82,83,108].

Furthermore, research has demonstrated that HOTAIR functions as a potent oncogene in various depressive disorders by modulating chromatin dynamics. Therefore, it is considered a key regulator of epigenetic regulation. HOTAIR’s role extends to ANRASSF1, which is jointly recruited by two expressions and cell proliferation by the polycomb repressive complex 2 (PRC2) at the majority of gene promoters [23]. Another example is NEAT1, which controls genes within signaling pathways by interacting with distant regulatory elements that do not necessarily depend on the effectiveness of these genes for their regulation. In summary, these examples support the theory that lncRNAs play diverse roles in epigenetic processes ranging from the recruitment of histone-modifying enzymes to the modulation of gene expression through the regulation of chromatin structure. Understanding the mechanisms by which lncRNAs contribute to various biological processes and diseases is crucial to understanding their significance in epigenetic processes.

### 3.4. Future Directions

In conclusion, ncRNAs, including miRNAs and lncRNAs, play crucial roles in the complex landscape of depression. Dysregulation of these ncRNAs in depressive states provides valuable insight into the molecular mechanisms underlying this disorder. Further exploration of ncRNA dysregulation in depression and functional studies to unravel its precise mechanisms of action will deepen our understanding of this disorder and potentially lead to innovative therapeutic interventions, as shown in Table 1. Addressing delivery challenges and understanding their specific functional roles are vital for translating the therapeutic potential of ncRNAs into effective treatment for depression. Continued scientific inquiry and interdisciplinary collaboration are essential to advance our knowledge and provide new hope for individuals with depression [109].

## 4. Dysregulation of Non-Coding RNAs in Depression

**Figure 4:**
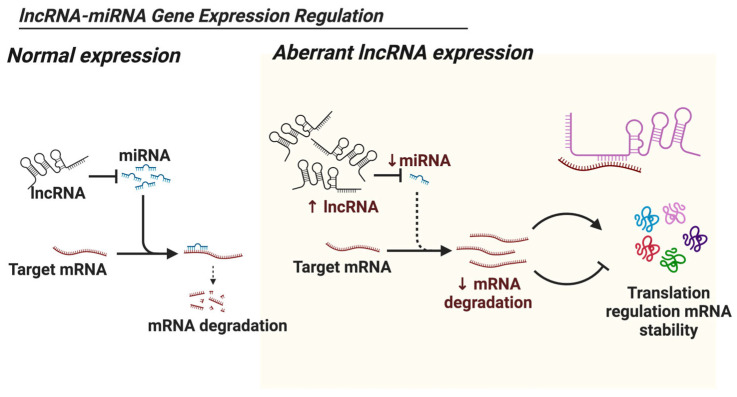
Schematic illustration of the regulatory mechanism of lncRNA-miRNA interactions in gene expression, highlighting the dysregulation observed in aberrant lncRNA expression. Specifically, it depicts the upregulation of lncRNAs, leading to decreased miRNA expression levels, subsequently resulting in reduced mRNA degradation and altered gene expression profiles compared with normal conditions. Images were generated using Biorender.com.

For example, many miRNAs reported to be dysregulated in brain regions implicated in depression are absent in genes related to neuroplasticity and synaptic function, as shown in Figure 4. Noteworthy examples include miR-16, miR-135a, and miR-let-7, which exhibit altered expression levels in depression and have been associated with the regulation of key genes [135,136]. The lncRNAs HOTAIR and MALAT1 are dysregulated in depression and have been implicated in the modulation of gene expression and cellular processes relevant to depression [81,137]. Non-coding RNAs serve as crucial regulatory elements in nearly all biological processes in the brain. Furthermore, neurotransmission system-related genes, such as those for serotonin and dopamine, are targeted by ncRNAs, mostly miRNAs [138].

### 4.1. Functional Consequences

Altered miRNA expression may lead to the dysregulation of neuroplasticity and synaptic connectivity, thereby contributing to the pathophysiology of depression. LncRNAs that have experienced dysregulation can engage with complexes that modify chromatin, thereby influencing epigenetic control of gene expression. Understanding the functional outcomes stemming from the disruption of ncRNA regulation offers vital perspectives on the molecular mechanisms underlying depression [139,140].

Deregulated ncRNAs can induce epigenetic changes through methylation, altering brain function, and potentially lead to neuronal cell death. Dysregulated ncRNAs can also serve as sensitive biomarkers and have been implicated in neurodegenerative diseases, including depression, thereby offering new diagnostic prospects. Long non-coding RNAs have been identified as potential regulators of cellular metabolism in non-communicable diseases, further highlighting their role in modulating cell functions [141].

### 4.2. Impact of Non-Coding RNAs on Gene Expression

By modulating the expression of target genes, ncRNAs can exert profound effects on cellular processes associated with depression such as neuroplasticity, neuroinflammation, and stress responses [105]. Various strategies, such as the use of antisense oligonucleotides or viral vectors, have been explored to target ncRNAs for therapeutic interventions. These strategies have shown efficacy in preclinical studies and have the potential to be translated into clinical applications [142]. However, challenges and limitations exist in the therapeutic targeting of ncRNAs. The efficient and targeted delivery of ncRNAs to specific brain regions is a primary obstacle, necessitating the development of innovative delivery methods. Off-target effects and unintended consequences of altering ncRNA expression must be addressed to ensure the safety and efficacy of therapeutic interventions [143].

Recent studies have demonstrated the potential of ncRNAs as diagnostic and therapeutic biomarkers for depression [118]. These studies have proposed that the interaction between N6-methyladenosine and non-coding RNAs is involved in the regulation of depression-related gene expression. This interaction highlights the role of m6A and ncRNAs in the development and progression of depressive disorders, and suggests potential targets for therapeutic intervention [144,145]. The identification of specific lncRNA-mRNA modules through co-expression network modeling revealed their involvement in stress-induced depression, emphasizing the active role of ncRNAs in altering the molecular phenotypes associated with depression [146]. Targeting specific ncRNAs with altered expression levels during the pathophysiology of this disorder may provide novel avenues for depression treatment [147,148].

The primary objective of the present study was to concentrate on ncRNAs while simultaneously considering the following key areas. While all three epigenetic mechanisms are relevant to depression, more recent work has been done on DNA methylation rather than histone modifications. In addition, ncRNAs have been demonstrated to be potential diagnostic biomarkers and therapeutic targets, with exciting possibilities for clinical applications in almost every recent study of depression. The implications of ncRNAs are of particular interest. In recent years, there has been an immediate increase in studies on ncRNAs and depression, providing significant information to researchers. Moreover, ncRNAs have great potential as novel diagnostic biomarkers and therapeutic targets, with exciting possibilities for clinical applications. Finally, the complex regulatory networks of ncRNAs involved in processes related to depression provide unique insights into the molecular mechanisms underlying this disorder. However, it is of interest to underline that DNA methylation and histone modification play key roles in research on depression.

## 5. Environmental Influences on Epigenetic Modifications

Early life experiences play a pivotal role in shaping an individual’s development and long-term health outcomes. Recent evidence has shed light on how these experiences can imprint lasting changes in the epigenome, leading to enduring alterations in the gene expression patterns. In this section, we critically examine the influence of early life experiences on epigenetic modifications to elucidate how environmental factors affect an individual’s epigenetic landscape [149].

Environmental factors, such as diet, chemical exposure, and lifestyle, have been found to potentially cause epigenetic changes associated with the development of depression [150]. In addition to genetic factors, the role of epigenetic modifications in depression is further emphasized by their connection to behavioral stress responses, highlighting the importance of epigenetics in understanding depression [151].

### 5.1. Prenatal Environment

The prenatal period represents a critical phase of development, during which the developing fetus is susceptible to environmental influences. The epigenetic terrain of the developing fetus can be molded significantly by factors such as maternal nutrition, stress levels, exposure to harmful substances, and the mental well-being of the mother [152]. A case in point is maternal nutrition, which has been linked to epigenetic alterations that govern genes associated with growth, metabolism, and neurodevelopment. Insufficient or uneven maternal nutrition can trigger shifts in DNA methylation and histone modifications in offspring, potentially shaping their health in the long term and predisposing them to diseases. Importantly, research has shown that folate intake by the mother influences DNA methylation patterns of genes associated with neurodevelopment, highlighting the significance of adequate nutrition during pregnancy [153]. Similarly, maternal stress experienced during pregnancy has been associated with epigenetic modifications in genes related to stress response mechanisms, such as those in the hypothalamic–pituitary–adrenal (HPA) axis. Stress encountered before birth can affect the DNA methylation patterns of genes responsible for regulating stress, potentially leading to modified stress reactivity in the offspring. Maternal anxiety during pregnancy has been associated with alterations in DNA methylation of stress-associated genes, including the glucocorticoid receptor gene (NR3C1), which could play a role in lasting changes in stress regulation [154]. Moreover, instances of exposure to environmental toxins, including substances such as tobacco smoke, air pollutants, or chemicals that disrupt the endocrine system, have been linked to epigenetic changes that occur during fetal development. Striking illustrations encompass prenatal exposure to cigarette smoke, which has been correlated with alterations in the DNA methylation of genes associated with lung development and immune system function [155].

### 5.2. Early Life Stress

Early life stress, which encompasses adverse childhood experiences (ACEs), can impose deep-seated and lasting effects on the developing brain and epigenome. For example, research has revealed shifts in DNA methylation within genes pertinent to the stress response, such as the FK506-binding protein five gene (FKBP5), among individuals who encounter early life stress. Epigenetic modifications that occur within genes linked to stress, such as NR3C1 and corticotropin-releasing hormone (CRH), have been correlated with modifications in stress responsiveness and an elevated predisposition to stress-associated disorders [8].

Chronic and repeated stressors can disrupt the regulation of the hypothalamic–pituitary–adrenal (HPA) axis and autonomic nervous system, leading to experience-dependent changes in the brain [27].

### 5.3. Parental Care

**Figure 5:**
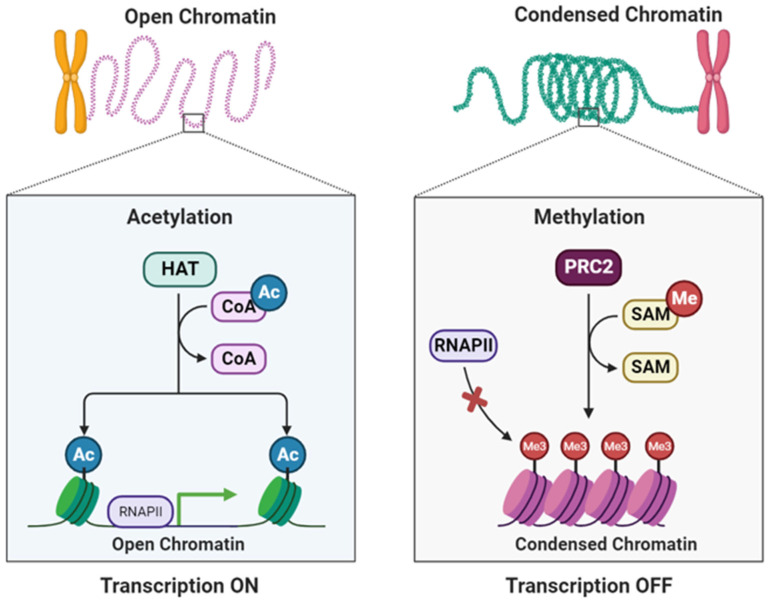
Dynamic chromatin regulation during transcription. This illustrates the influence of the chromatin state on transcriptional activity. Open chromatin, marked by acetylation (Ac), facilitates transcriptional activation, whereas condensed chromatin, characterized by methylation (Me), leads to transcriptional repression. Understanding the interplay between these epigenetic modifications provides insights into the nuanced regulatory mechanisms governing gene expression. Abbreviations used in the figure include histone acetyltransferase (HAT), acetylation (Ac), CoA (coenzyme A), RNAPII (RNA polymerase II), PRC2 (polycomb repressive complex 2), SAM (S-adenosylmethionine), Me (methylation), and Me3 (trimethylation). Images were generated using Biorender.com.

Early life experiences have a profound impact on the epigenome, which in turn influences long-term health outcomes [156]. For example, studies have found DNA methylation alterations in genes such as the oxytocin receptor gene (OXTR) and brain-derived neurotrophic factor gene (BDNF) among individuals exposed to early life adversity [157]. Multiple studies have established a connection between childhood trauma and epigenetic modifications, particularly in the genes related to stress regulation, emotional processing, and neuroplasticity. These alterations in DNA methylation, along with disrupted epigenetic control of genes, such as BDNF and SLC6A4, suggest a possible link between early life experiences, epigenetic changes, and increased vulnerability to depression. Longitudinal studies have demonstrated the intricate and multifaceted nature of mechanisms connecting childhood trauma, epigenetic modifications, and depression. These studies found that epigenetic changes resulting from childhood trauma can persist into adulthood, leading to altered gene expression patterns and an increased risk of developing depression [158]. Future studies should investigate other epigenetic indicators, such as histone modifications and non-coding RNAs, to gain a more comprehensive understanding of the epigenetic landscape in individuals with a history of childhood trauma [109,159]. Furthermore, changes in histone modifications can be induced by environmental signals such as stress or exposure to enriched environments, which affect the accessibility of chromatin and gene expression patterns, as shown in Figure 5 [160].

Moreover, adjustments to the activity of enzymes involved in epigenetic regulation, along with the influence of stress-related signaling pathways, such as the hypothalamic–pituitary–adrenal (HPA) axis and the sympathetic nervous system, contribute to the complex interplay of mechanisms that shape an individual’s epigenome and susceptibility to various health outcomes, including mental health disorders [132,133,161].

### 5.4. Stress and Depression

This section aims to elucidate the relationship between chronic stress, depression, and epigenetic modifications and reviews pertinent studies investigating stress-induced epigenetic changes associated with depression [14]. Epigenetic alterations, including DNA methylation, histone modifications, and non-coding RNAs, play crucial roles in mediating this intricate interplay, influencing genes linked to stress regulation, neuroplasticity, and neurotransmitter signaling [162]. Chronic stress has been shown to induce DNA methylation alterations within stress-responsive genes, such as the glucocorticoid receptor gene (NR3C1), disrupting its expression and the effective negative feedback mechanism of the hypothalamic–pituitary–adrenal (HPA) axis [163]. Nonetheless, further investigation and validation are required to elucidate the precise mechanisms through which these modifications contribute to the onset and perpetuation of depression [10]. Among these, brain-derived neurotrophic factor (BDNF) has garnered significant attention because of its crucial role in neuronal survival and synaptic plasticity. However, it is essential to recognize that other factors, including genetic variations and environmental interactions, can also influence BDNF regulation, necessitating further research on the multifaceted nature of this relationship [164]. Although the specific mechanisms linking these genes and pathways to epigenetic changes are still under investigation, their involvement underscores the intricate interplay between epigenetics, stress, and depression [150].

## 6. Epigenetics and Treatment Approaches

Substantial progress in some measures, portraying how the molecular basis of depression has been forged in the past few years, has revealed various underlying aspects. The prime focus has increased further toward epigenetics as another promising avenue that describes gene expression exerting an influence on neuronal circuitry, but it does not give rise to changes in the DNA sequence [165]. One such identified target is epigenetic DNA methylation, which has also been developed into a process involving the accumulation of methyl groups onto DNA buildups and has now been found to be among the leading mechanisms participating in the pathophysiology of depression. Other genes that are aberrantly methylated and can be harnessed include several that promote neuroplasticity and synaptic functioning in the management of this malaise. Histone modification is an important mechanism due to the fact it initiates gene expression by causing changes in the chromatin structure. Other echoed approaches for developing new classes of antidepressants have been based on the modulated activity of the enzymes responsible for histone modification. MicroRNAs (miRNAs), a small family of RNA elements, have also been identified as putative new therapeutic targets within regimens for the treatment of depression [48,166]. The analysis of epigenetic profiles may enable the identification of specific biomarkers associated with treatment response or vulnerability to antidepressant drugs. This knowledge can inform treatment selection and facilitate a personalized and effective approach to managing depression. Monitoring changes in epigenetic profiles during treatment may offer insights into treatment efficacy and guide therapeutic adjustments [57]. Moreover, personalized medicine approaches may be extended to preventive interventions by identifying early markers of vulnerability to depression based on epigenetic profiling [48]. The field of epigenetics presents a wealth of opportunities for the development of novel antidepressant drugs and the implementation of personalized medicine in depression treatment.

Focusing on pivotal EMs, such as DNA methylation, histone modifications, and miRNA regulation, has the potential to drive the development of innovative therapeutic strategies. By integrating the insights gained from epigenetics into treatment approaches, the possibility arises to elevate patient outcomes and facilitate tailored interventions that cater to individual needs [165]. Epigenetic biomarkers may serve as predictors of treatment response and aid treatment selection. Ongoing investigations are exploring epigenetic modulators as potential antidepressant drugs to reverse the abnormal epigenetic marks associated with depression [50]. Exploring epigenetic effects in psychotherapy and behavioral interventions yields promising insights into their therapeutic benefits for depression. Integrating epigenetic knowledge into therapeutic strategies can personalize treatments, identify predictive biomarkers, and develop novel interventions targeting specific epigenetic modifications [14]. However, ethical considerations must be carefully addressed when integrating epigenetic information into clinical practice [167], in addition to ongoing research. The above-mentioned epigenetic studies are surely set for potentially reforming the whole scenario of the psychiatry field, and they provide leading-edge responses regarding how to effectively manage depression and well-being in a patient through enhancement and making psychiatric treatment more personalized for everyone.

The discovery of small-molecule inhibitors that target epigenetic regulators has ushered in a new era of epigenetic-targeted therapies for hematologic malignancies and solid tumors [168]. With refined drug delivery methods, drugs have been effectively directed to their intended sites of action, resulting in improved therapeutic outcomes [169]. Thus, the development of epigenetic drugs has emerged as a novel treatment approach for various major illnesses, including cancer, cardiovascular disorders, and brain disorders [170]. Epigenetic therapy has been designated as a means of addressing mental health disorders [171]. Although a detailed discussion of epigenetic modifications as therapeutic targets is beyond the scope of our analysis, they have demonstrated the potential for understanding disease risk, progression, and clinical response, particularly when combined with cytotoxic agents to enhance clinical outcomes [172]. Targeting calcium signaling through epigenetic pathways has been a focus of interest in attempts to modulate gene expression in the context of depression. This underlines how epigenetic mechanisms, such as DNA methylation and histone modifications, may play a role in neuronal plasticity and stress response pathways, which are central to the pathophysiology of depressive disorders. To understand how these epigenetic changes influence calcium signaling, new therapeutic strategies aimed at the reactivation of beneficial gene expression patterns disrupted in depression have been developed [173]. Based on the literature described above, it can be asserted that epigenetic therapy holds significant potential in diverse areas of medicine, ranging from cancer care to cognitive and mental health disorders [174]. Targeted and specific utilization of epigenetic regulators for therapeutic intervention provides a pathway for personalized treatment strategies. The effectiveness of treatments involving epigenetic modifications has highlighted the importance of researchers, clinicians, and current approaches for the development of innovative therapies for complex diseases.

Some of the most promising strategies for combating central nervous system disorders, including depression, are epigenetic drugs, generally referred to as epidrugs. These drugs target several mechanisms of epigenetic regulation, such as histone deacetylases, DNA methyltransferases, bromodomains, and ten-eleven translocation proteins, which eventually affect the expression of key mediators involved in depression, including neuroimmune inflammation factors, neurotrophins, ion channels, and pathoproteins such as β-amyloid and tau protein [175].

Research on the mechanisms underlying chromatin regulation in depression has increasingly focused on genome-wide approaches. Such a shift is necessary to truly understand the epigenetic landscape of depression; perhaps more importantly, it sets the eventual target for using transcriptional and epigenomic data from human postmortem tissue to develop better strategies for the treatment of depression [176]. Pharmacogenetic studies have provided evidence of the role of genetic polymorphisms associated with metabolizing enzymes and transporters in response to antidepressant and anxiolytic therapies [177].

Furthermore, human epigenetic therapeutics require insight into the landscape of human epigenetic enzymes and modulators. Databases such as HEMD, the Human Epigenetic Enzyme and Modulator Database, provide detailed information for researchers and clinicians who aim to identify potential epigenetic targets for therapeutic intervention [178]. DNA methylation inhibitors have been demonstrated to exert antidepressant-like effects in preclinical studies as they lower DNA methylation and increase the expression of BDNF in these encephalic regions related to depression. These results emphasize that targeting DNA methylation pathways may be a potential therapy for depression [179].

### Clinical Applications of Epigenetic Research

Recent epigenetic advances have opened new avenues for the improvement of patient care. This section summarizes the translation of epigenetic discoveries into clinical practice. It covers diagnosis, treatment selection, and monitoring responses. The early detection of the disease is one of the most important challenges in medicine. Epigenetic biomarkers may provide answers to this question. Among them are the DNA methylation patterns in cell-free DNA, which have recently been suggested to potentially be used for the early detection of depression [180]. Epigenetic changes due to host microbiota have also been implicated in depressive disorders, providing new hope for nutritional and probiotic treatments. Interventions that target the gut microbiota, such as modulation by probiotics, dietary interventions, and fecal microbiota transplantation, have been found to be effective in sculpting an epigenetic landscape toward the improvement of depressive symptoms in affected individuals [179,181]

Many studies have identified signatures of methylation related to depression, which are epigenetic patterns that are seen in multiple tissues, such as blood and brain samples, and help point to some subtypes or symptom profiles of depressive disorders. Clearly, researchers are now studying the utility of these methylation signatures in improving diagnostic accuracy and predicting treatment responses, and probably defining those people at risk for developing depression [182]. These tests are being tested against existing screens in current trials [183,184,185]. Currently, they are being tested for their effectiveness and cost-effectiveness. In treatment selection, epigenetic profiles can inform treatment choices in a variety of conditions. For example, gene methylation is linked to the response of some leukemias to chemotherapy [186]. Similar approaches have already been taken for other psychiatric and neurological disorders, such as schizophrenia, bipolar disorder, and Alzheimer’s disease. This will provide a basis for tailoring treatment strategies in patients with depression based on their epigenetic profiles. The research underway in this area is mainly concerned with the development of robust epigenetic biomarkers and their integration into practice to enhance the management of depressive disorders [186,187,188]. Furthermore, monitoring treatment responses using epigenetic markers can reveal the effectiveness of treatment.

Another aspect of the proposed treatment strategy is the integration of epigenetic therapies. Drugs based on EMs have been used in clinical applications. They have been approved as inhibitors of DNA methyltransferases and histone deacetylases [189]. Further research is required to confirm these results. This will help determine whether the combination of epigenetic drugs with other therapies is promising. Previous studies have shown that epigenetic drugs can be used to achieve this goal. They can increase the sensitivity of tumors to immunotherapy and classic chemotherapy. This is an excellent example of a potential combination treatment [190,191]. Implementation in clinical practice presents several challenges. First, standard test procedures were required. These results are consistent with those obtained in different laboratories. Second, health care providers should be educated on the interpretation and use of epigenetic data. Thirdly, clear clinical guidelines are required for epigenetic testing and treatment. Finally, ethical concerns must be addressed [192,193].

Several trials are currently in progress. They have studied epigenetic methods and their impact on patient outcomes and health care costs. As data from these trials emerge, they will shape how epigenetics is used in clinical practice. This will likely open new ways to personalize prevention, diagnosis, and treatment for patients [194,195,196].

## 7. Recent Advances in Epigenetic Technologies

Advances in epigenetic technologies have progressed rapidly. This section explores three key areas of development. These include CRISPR-based tools for editing the epigenome, single-cell epigenomics, and sequencing methods.

The new CRISPR-based methods have expanded the scope of epigenetic editing. The CRISPR-Cas9 system can be employed to modify genes and alter DNA methylation, histone modifications, and chromatin structure [197]. This system offers greater flexibility and ease of use than older techniques such as zinc finger proteins and Transcription Activator-Like Effector Nucleases (TALENs). Its applications extend to the exploration of gene regulation and the search for treatments [198].

Single-cell epigenomics represents the development of new methods for studying cells at an individual level [199]. These methods focus on examining DNA methylation, chromatin accessibility, and histone modifications, and have proven to be highly beneficial in fields such as developmental biology and cancer research. Studies on the differences highlight the importance of single-cell epigenetics. Furthermore, combining single-cell epigenetics with other omics data can provide a comprehensive view of cell states and behaviors and the complexities of cellular processes [200,201,202,203]. Single-cell epigenomics has greatly advanced our understanding of cellular biology.

Advances in sequencing technology have facilitated the enhancement of epigenetic profiling. Third-generation platforms such as PacBio and Oxford Nanopore provide long-read methods that are better equipped to capture large-scale epigenetic patterns than short-read methods [204,205]. Furthermore, improvements in bisulfite sequencing and the emergence of new methods such as TAPS and EM-seq have increased the accuracy of DNA methylation mapping. Spatial epigenomic techniques have revealed variations in epigenetic modifications across tissues and organs [206,207]. These advancements have provided a clearer understanding of the role of epigenetics in both development and disease [208,209].

## 8. Summary and Conclusions

This study explored epigenetics and its relationship with depression, with a specific focus on its potential application in the development of new antidepressant drugs and personalized medical strategies. Much of this research has future implications. Examples include assessment and intervention with environmental factors impacting epigenetics, making lifestyle recommendations, using epigenetic testing when available, and offering psychotherapies known to instigate beneficial epigenetic changes, to explain the timing of treatment to a patient. These steps help reduce the translational gap between current research and clinical practice.

Epigenetics is a fundamental discipline that aids in understanding the biological mechanisms underlying depression and provides valuable insights into its molecular foundation. Recognizing the intricate interplay between genetic and environmental factors is crucial for understanding the pathophysiology of depression and for devising innovative treatment strategies.

Epigenetic markers include DNA methylation and histone modifications, which are important regulators of depression-associated gene expression. Aberrant epigenetic marks have been detected in patients with depression. For instance, hypermethylation of the BDNF gene promoter has been reported to be associated with the reduced expression of this critical neurotrophic factor during depressive behavior. These epigenetic markers can be used to identify precise molecular targets for intervention.

Very little is known about the technologies used in epigenetic research. Future research should include longitudinal studies and experimental designs to establish the causal connections between epigenetic changes and depression. The main objective of personalized medicine is to identify robust epigenetic biomarkers that can guide treatment selection, predict outcomes, and facilitate the development of tailored interventions. By exploring the synergistic effects of epigenetic approaches and other treatments such as pharmacotherapy and neuromodulation techniques, optimal therapeutic outcomes can be achieved [165].

The incorporation of epigenetic approaches into personalized treatment for depression presents promising prospects for improving patient outcomes. Understanding the dynamic interplay between genetic and environmental factors can lead to the development of targeted interventions to address the specific molecular pathways that contribute to depression. However, in pursuit of progress, ethical considerations related to the use of epigenetic information must be acknowledged. Ensuring patients’ well-being and safeguarding their rights necessitates the responsible and thoughtful implementation of epigenetic approaches. In conclusion, epigenetics has emerged as a profound source of insight into the biology of depression. By comprehending epigenetic changes associated with depression and integrating this knowledge into therapeutic strategies, ongoing research in this dynamic field undoubtedly holds the promise of contributing to the improvement of the lives of individuals affected by depression and shaping the future of psychiatric care.

## Figures and Tables

**Table 1:** This table provides a comprehensive summary of the relationships between depression, major depressive disorder (MDD), dysthymic disorder, and depression in bipolar disorder and various EMs, including DNA methylation, histone modifications, non-coding RNAs (miRNAs and lncRNAs), chromatin remodeling, and DNA hydroxy methylation.

Epigenetic Mechanisms (EM)	Relationship to Depression	Relevant Studies
**DNA Methylation**	Altered DNA methylation patterns in genes associated with neurodevelopment and neurotransmission have been linked to depression susceptibility and treatment response.	- Investigation of DNA methylation in the serotonin transporter gene (SLC6A4) and its association with depression risk and MDD and antidepressant response. The focus appears to be on understanding the relationships between MDD, childhood trauma, and biological factors such as DNA methylation and hippocampal volume [110]. - Study on DNA methylation changes in brain-derived neurotrophic factor (BDNF) and their role in depression pathophysiology [111]. - Genome-wide DNA methylation profiling in MDD, identifying epigenetic biomarkers and biological pathways involved in the disorder. Methylation levels of 14 DMRs in genes predisposing toward major depressive disorder were positively associated with depression scores, giving a possible mechanism for how methylation affects depression. Genes of interest were the following: BMP2, PRDM7, KCNIP1, GRIK2, TPRN2, GATA2, HELZ2, and ZNF624 [112,113].
**Histone Modifications**	Dysregulation of histone-modifying enzymes has been observed in depression, affecting genes involved in synaptic plasticity and stress response.	- The role of circadian genes and histone modifications in MDD such as those affecting sleep, temperature, hormonal secretions, and mood are associated with MDD and antidepressant treatment [114]. - Investigation of chromatin modifications and their impact on cognitive behaviors in depression [12]. - Epigenetic regulation of genes implicated in synaptic function and neuronal plasticity in MDD [45].
**Non-coding RNAs (miRNAs)**	Dysregulation of miRNAs has been associated with neuroinflammation, synaptic plasticity, and stress response pathways, making them potential diagnostic biomarkers and therapeutic targets for depression.	- MicroRNAs in MDD, depression, and suicide disorder [115]. - Altered miRNA expression in peripheral blood mononuclear cells in depression patients, revealing potential circulating biomarkers [116].
**Long Non-coding RNAs (lncRNAs)**	Aberrant expression of lncRNAs has been implicated in depression, modulating gene expression and affecting key pathways in the brain.	- Comprehensive profiling of lncRNA expression in postmortem brain tissues of depression patients, revealing potential therapeutic targets [117,118]. - Meta-analysis of lncRNA expression patterns in blood samples of patients with MDD [103].
**Chromatin Remodeling**	Altered chromatin remodeling complexes have been associated with depression, influencing the accessibility of genes involved in neuroplasticity and stress response.	- The roles of histone acetylation, DNA methylation, and non-coding RNA. Behavioral response to stress, depressive behaviors, and response to antidepressants [119]. - Chromatin medication and their cognitive behaviors in depression [120]. - Identification of altered chromatin accessibility in MDD patient-derived neurons, providing insights into the transcriptional dysregulation by studying open and closed chromatin states in the brain, with emphasis on neuropsychiatric disorders [121]. - Altered chromatin accessibility in MDD patient-derived neurons [122]
**DNA Hydroxy methylation**	Changes in DNA hydroxy methylation have been observed in depression, potentially influencing gene expression and neurodevelopmental processes.	- Investigation of DNA hydroxy methylation dynamics in depression using postmortem brain tissues [123]. - Altered DNA hydroxy methylation patterns in peripheral blood samples of depression patients [124]. - The role of DNA hydroxy methylation in depression susceptibility and treatment response [125].
**RNA Editing**	Dysregulation of RNA editing enzymes has been implicated in depression, leading to altered transcriptomes and neurobiological processes.	- RNA editing landscape in MDD patient brains, highlighting potential editing sites in genes relevant to depression [126]. - Study on the impact of RNA editing adenosine to inosine (A-to-I) RNA editing and m6A methylations on MDD [126]. - Altered RNA editing profiles in peripheral blood samples of patients with treatment-resistant depression and bipolar disorder. The association of RNA editing variant modifications with depression subtypes [127].
**Chromosomal Conformation**	Changes in chromosomal conformation have been associated with depression, affecting gene interactions and regulatory networks in the brain.	- Hi-C analysis of chromosomal interactions in MDD, dysthymic disorder, and depression in bipolar disorder patient brain tissues, identifying altered 3D chromatin organization [128]. - Investigation of chromosomal interactions and their role in gene regulation in a mouse model of depression [129]. - Altered chromosomal conformation associated with genes involved in synaptic function and depressive behaviors in MDD [130].
**Transgenerational Epigenetic Inheritance**	Epigenetic changes in parental germ cells have been implicated in the risk of depression in offspring, suggesting transgenerational epigenetic inheritance.	- Investigation of transgenerational effects of stress-induced DNA methylation changes on chronic stress susceptibility in rat offspring [131]. - Human study exploring the potential impact of parental early life stress on DNA methylation patterns in MDD, dysthymic disorder, and depression in bipolar disorder patients and their children [132,133]. - Evidence for transgenerational epigenetic inheritance in depression and its underlying depression mechanisms [134].

## Data Availability

No data were generated during the preparation of the manuscript.
